# Fertility‐Related Concerns in Survivors of Childhood Cancer: A Systematic Review

**DOI:** 10.1002/cam4.71045

**Published:** 2025-07-14

**Authors:** Pauline Holmer, Martina Ospelt, Gisela Michel, Vicky Lehmann, Fiona S. M. Schulte

**Affiliations:** ^1^ Faculty of Health Sciences and Medicine, University of Lucerne Lucerne Switzerland; ^2^ Department of Medical Psychology Amsterdam University Medical Center/University of Amsterdam Amsterdam the Netherlands; ^3^ Cancer Center Amsterdam Amsterdam the Netherlands; ^4^ Department of Oncology, Division of Psychosocial Oncology University of Calgary Calgary Canada; ^5^ Faculty of Human Sciences, Department of Inclusive Education University of Potsdam Potsdam Germany

**Keywords:** cancer, cancer survivors, childhood cancer, concerns, fertility, oncology, reproduction

## Abstract

**Objective:**

Childhood cancer survivors are at increased risk for treatment‐related fertility impairment and subsequent fertility‐related concerns. However, there is currently no consensus on how to define, assess, and address these concerns in clinical practice. Therefore, we aimed to answer the following questions: (1) How are fertility‐related concerns assessed and defined? (2) What types of fertility‐related concerns are reported by survivors, and how common are they? (3) What clinical implications are proposed in the literature?

**Methods:**

We systematically searched the databases PubMed, PsycINFO, MEDLINE, CINAHL, and Embase for peer‐reviewed publications on fertility‐related concerns in survivors of childhood cancer published between January 1990 and November 2023. The search yielded 2077 potentially relevant articles, of which eleven met inclusion criteria and were included. Furthermore, we screened the reference lists of included studies, leading to a total of *N* = 25 studies included in the narrative synthesis.

**Results:**

We identified considerable variation in the assessment, description, and definition of fertility‐related concerns. Survivors described fertility‐related concerns in terms of their personal and reproductive health, the health of their (future) children, and dating and romantic relationships. Concerns were reported by 9.5% to 65% of survivors in quantitative studies. Suggestions for clinical implications included that different healthcare providers could use individual or group strategies to address fertility‐related concerns. They should focus on patient reports rather than parent‐proxy reports and assess individual fertility‐related preferences and attitudes without making assumptions.

**Conclusions:**

Fertility‐related concerns are common among childhood cancer survivors and need to be addressed by care providers and researchers in the field. To do so effectively, we need more consistency in assessing, labeling, and defining these concerns.

## 1 Introduction

With significantly improved survival rates for childhood cancer, the number of survivors continues to grow worldwide [1]. As a result, improving the quality of life of survivors has become a priority. Treatment‐related morbidity can extend well into survivorship and lead to a substantial burden that diminishes well‐being [2]. Various treatment modalities, such as gonadotoxic chemotherapy or abdominal surgery, can lead to later fertility impairment in both male and female survivors [3, 4]. Beyond actual infertility, defined as the failure to achieve a successful pregnancy after 12 months or more of regular, unprotected sexual intercourse or due to an impairment of a person's capacity to reproduce either as an individual or with their partner [5], childhood cancer survivors are at risk for various fertility‐related problems. These include impaired spermatogenesis, premature ovarian insufficiency, miscarriages, preterm delivery, or having a low‐birth‐weight baby [3, 4, 6, 7, 8, 9, 10]. Given the risk for treatment‐related fertility problems, survivors may have fertility‐related concerns [11]. Getting a better understanding of these concerns is essential in order to be able to address them appropriately.

Previous research indicates that most survivors of childhood cancer want to have (biological) children later in life [12], which is why compromised reproductive function can lead to high levels of distress [3]. Dealing with (potential) infertility presents challenges in many areas of young survivors' lives, including coping with developmental tasks (e.g., becoming a parent), forming an identity, engaging in romantic relationships, and finding meaning in their lives that is not defined by cancer [13].

While guidelines exist for fertility preservation at the time of diagnosis [3, 4] and related communication and ethical aspects [14], there is insufficient consensus on how to assess and address survivors' concerns later in life. The current lack of agreement on the assessment, definition, and clinical management of *fertility‐related concerns* in childhood cancer survivors highlights the need for a systematic review of existing literature. In this systematic review, we aimed to address the following questions: (1) How are fertility‐related concerns assessed and defined? (2) What types of fertility‐related concerns do survivors describe, and how prevalent are they? (3) What clinical implications are proposed by the authors?

## 2 Methods

### 2.1 Literature Search

This review is based on the reporting guidelines described in the Preferred Reporting Items for Systematic Reviews and Meta‐Analysis (PRISMA) checklist (Supporting Information S1: Appendix A). It was registered on PROSPERO (No. CRD42023485113). On November 21, 2023, we systematically searched the databases PubMed, PsycINFO, MEDLINE, CINAHL, and Embase. We used the search blocks for *childhood cancer*, *survivors*, *fertility*, and *concerns* (Supporting Information S1: Appendix B). We used the search blocks for childhood cancer and survivors from previous publications [15, 16] and developed the ones on fertility and concerns for this systematic review. All search blocks were adapted for each database.

### 2.2 Selection Criteria

We included original research papers that presented self‐reported fertility‐related concerns of childhood cancer survivors. The detailed selection criteria are displayed in Table 1.

**TABLE 1:** Study selection criteria.

	Inclusion criteria	Exclusion criteria
Participants	At least 3 participants Cancer diagnosis (≥ 75% of the sample, or separate subgroup analyses) Age at diagnosis < 21 years (≥ 75% of the sample, or separate analyses) Time since diagnosis ≥ 2 years (≥ 50% of the sample, or separate analyses) Off‐treatment at time of study (≥ 75% of the sample, or separate analyses) At least 18 years old at time of study (≥ 75% of the sample, or separate analyses)	
Types of study	Published since 1990 Published in a peer‐reviewed journal Human subjects All study designs assessing survivors' perceptions	Case reports Papers not presenting original data (e.g., systematic reviews)
Main outcome	Fertility‐related concerns/worries/fears/uncertainties	Fertility status/testing/biological factors (unless combined with fertility‐related concerns)
Language	English	

*Note:* The age cutoffs were selected to reflect broad international definitions of childhood cancer (< 21 years at diagnosis), to focus on the post‐treatment survivorship phase (≥ 75% of the sample), and to ensure participants were legal adults likely capable of informed consent and developmentally able to reflect on fertility and parenthood (≥ 18 years old at time of study).

### 2.3 Study Screening

All identified records from the systematic search were entered into a Zotero bibliography, where duplicates were automatically removed. All remaining titles and abstracts were then independently screened by two reviewers (PH and MO) using the free web tool Rayyan (https://rayyan.ai/). We proceeded with the full text screening in a separate Rayyan bibliography, where again, two reviewers (PH and MO) screened the full texts independently. If an article that seemed to fit based on title and abstract was excluded, the reason was provided. Disagreements were resolved through discussion (PH and MO). After selecting the appropriate studies based on their full text, their reference list was screened for more potentially relevant papers (PH).

### 2.4 Quality Assessment

The methodological quality of the included studies was rated using the Quality Assessment with Diverse Studies (QuADS) tool [17], which has demonstrated substantial reliability and validity. Thirteen items were used to assess the extent to which the publications covered common quality indicators on a scale from 0 (no mention at all) to 3 (complete). The maximum possible score was 39. Two reviewers (PH and MO) independently assessed the quality of each of the included articles.

### 2.5 Data Extraction and Analysis

Once all relevant studies were selected, key information was transferred to a predefined data extraction sheet. The data extraction sheet was used to collect information on (1) study characteristics; (2) assessment and definition of fertility‐related concerns; (3) prevalence of fertility‐related concerns; (4) facets/associated factors of fertility‐related concerns as described by survivors; and (5) clinical implications suggested by the authors. Extracted data was synthesized narratively.

## 3 Results

### 3.1 Study Selection

Our search yielded 2077 hits in all five databases. After removing duplicates, we screened 1173 titles/abstracts, 87 full texts, and finally included 11 studies. From all reference lists of these studies, we screened another 116 potentially relevant references and included an additional 14 studies, resulting in a final number of *N* = 25 included studies. Detailed information on the study selection process can be found in Supporting Information S1: Appendix C.

### 3.2 Study Characteristics

Most of the 25 included studies were from the US (*n* = 12), followed by the Netherlands (*n* = 4). Fertility‐related topics were the main outcome/one of the main outcomes in *n* = 14 studies. Most studies followed a qualitative approach (*n* = 14). In total, more than *N* = 4200 survivors of childhood cancer participated in all studies combined (Table 2).

**TABLE 2:** Overview of included studies, sorted by year of publication.

Title (study design)	Authors (year)^REF^	Country	Number of survivors	Sex/gender distribution	Mean age at diagnosis in years (SD/range)	Mean time since diagnosis in years (SD/range)	Mean age at study in years (SD/range)	Main outcome
Infertility and Identity: A Closer Look Into experiences of emerging young adult childhood cancer survivors (qualitative)	Sira et al. (2023) [13]	US	*N* = 6	F *n* = 5 M *n* = 1	NR	NR	23.2 (NR/18–29)	Yes
Positive and negative survivor‐specific psychosocial consequences of childhood cancer: the DCCSS‐LATER 2 psycho‐oncology study (quantitative)	Maas et al. (2023) [18]	NL	*N* = 1713	F 48.9% M 51.1%	6.8 (4.7/0.0–18.0)	29.2 (8.5/15.3–55.0)	36.0 (9.3/18.3–70.9)	No
Including a discussion forum in a web‐based intervention on fertility and sexuality following cancer – usage and content (qualitative)	Gottvall et al. (2022) [19]	SE	*N* = 322	F 64.6% M 35.4%	7.3 (5.5/NR)	21.2 (7.4/NR)	29 (5.8/NR)	Yes
A vulnerable age group: the impact of cancer on the psychosocial well‐being of young adult childhood cancer survivors (quantitative)	van Erp et al. (2021) [20]	NL	*N* = 151	F 61.6%	10.5 (4.5/0.4–17)	13.6 (3.8/6–27)	24.1 (3.6/18–30)	No
Facing the unknown: uncertain fertility in young adult survivors of childhood cancer (qualitative)	Newton et al. (2020) [11]	CA	*N* = 25	F *n* = 15 M *n* = 10	9 (NR/< 1–19)	NR	29 (NR/23–36)	Yes
Higher reproductive concerns associated with fertility consultation: a cross‐sectional study of young adult male cancer survivors (quantitative)	Drizin et al. (2020) [21]	US	*N* = 47	M 100%	NR (NR/1–34)	4.9 (5.1/1–17)	28.1 (4.9/18–35)	Yes
Romantic relationships and physical intimacy among survivors of childhood cancer (qualitative)	Nahata et al. (2020) [22]	US	*N* = 40	F 62.5% M 37.5%	11.1 (3.2/5–17)	18.4 (5.9/10–37)	29.8 (4.8/23–42)	Yes
The perceived impact of infertility on romantic relationships and singlehood among adult survivors of childhood cancer (qualitative)	Lehmann et al. (2019) [23]	US	*N* = 30	F 66.7% M 33.3%	11.8 (3.7/5–18)	18.0 (4.4/12–28)	29.8 (4.4/23–41)	Yes
Problems of reproductive function in survivors of childhood‐ and adolescent and young adult‐onset cancer revealed in a part of a national survey of Japan (quantitative)	Furui et al. (2018) [24]	JP	*N* = 76	F 58% M 42%	F 7.5 (4.8/NR) M 7.7 (4.2/NR)	NR	F 24.7 (6.2/NR) M 23.0 (5.6/NR)	Yes
Fertility issues in adolescent and young adult cancer survivors (qualitative)	Benedict et al. (2016) [25]	US	*N* = 43	F 58.1% M 41.9%	15.4 (1.5/14–19)	3.3 (2.2/0.9–9.3)	19.6 (2.8/19–20)	Yes
Primary gonadal insufficiency in male and female childhood cancer survivors in a long‐term follow‐up clinic (quantitative)	Gunn et al. (2016) [26]	AU	*N* = 54	M 61.5%	Median: 4.8 IQR: 3.0–9.7	NR	Median: 22.3 IQR: 18.2–25.7	No
Sexual dysfunction in young adult survivors of childhood cancer (qualitative)	Frederick et al. (2016) [27]	US	*N* = 22	F 54.5% M 45.5%	13.0 (4.6/1–20)	NR	22.6 (3.5/18–31)	No
Worries of childhood cancer survivors in young adulthood (qualitative)	Yi et al. (2016) [28]	KR	*N* = 28	F 46.4% M 53.6%	11.9 (2.9/5–17)	12.9 (5.9/6–25)	24.9 (4.1/20–36)	No
Fertility preservation preferences and perspectives among adult male survivors of childhood cancer and their parents (qualitative)	Stein et al. (2014) [29]	US	*N* = 15	M 100%	14 (NR/10–20)	22 (NR/9–35)	35 (NR/25–47)	Yes
Will I be able to have a baby? Results from online focus group discussions with childhood cancer survivors in Sweden (qualitative)	Nilsson et al. (2014) [30]	SE	*N* = 134	F 51% M 49%	Median: 8 (NR/0–17)	Median: 12 (NR/5–23)	Median: 21 (NR/16–24)	Yes
Impact of childhood cancer on emerging adult survivors' romantic relationships: A qualitative account	Thompson et al. (2013) [31]	US	*N* = 18	F 100%	7.4 (4.7/2–15)	NR	21.6 (2.2/19–25)	Yes
Psychometric evaluation of the impact of cancer (IOC‐CS) scale for young adult survivors of childhood cancer (quantitative)	Zebrack et al. (2010) [32]	US	*N* = 519	F 51.8% M 48.0%	11.3 (6.1/0–21)	15.4 (6.9/2–37)	26.7 (5.3/18–39)	No
Male and female experiences of having fertility matters raised alongside a cancer diagnosis during the teenage and young adult years (qualitative)	Crawshaw et al. (2009) [33]	GB	*N* = 38	F *n* = 21 M *n* = 17	Median:15 (NR/11–20)	Teenagers: Median: 3 (NR/1–6) Adults: Median: 7 (NR/2–15)	Median: 21 (NR/16–30)	Yes
Fertility issues for young adult survivors of childhood cancer (qualitative)	Zebrack et al. (2004) [34]	US	*N* = 32	F 44% M 56%	7.0 (NR/0–16)	17.1 (NR/7–36)	24.2 (NR/19–37)	Yes
Quality of life, self‐esteem and worries in young adult survivors of childhood cancer (quantitative)	Langeveld et al. (2004) [35]	NL	*N* = 400	F 45% M 55%	8 (4.6/NR)	NR	24 (4.9/NR)	No
Educational achievement, employment and living situation in long‐term young adult survivors of childhood cancer in the Netherlands (quantitative)	Langeveld et al. (2003) [36]	NL	*N* = 500	F 47% M 53%	8 (4.7/0–19)	NR	24 (5.1/16–49)	No
The impact of childhood cancer on adult survivors' interpersonal relationships (mixed‐methods)	Forsbach & Thompson (2003) [37]	US	*N* = 111	NR	NR	NR	24.8 (NR/18–45)	No
The psycho‐social impact of infertility on young male cancer survivors: A qualitative investigation (qualitative)	Green et al. (2003) [38]	GB	*N* = 15	M 100%	NR	NR	NR (NR/19–32)	Yes
Self‐Reported worries among long‐term survivors of childhood cancer and their peers (mixed‐methods)	Weigers et al. (1998) [39]	US	*N* = 228	M 33.8%	NR	NR	17.6 (NR/NR)	No
Psychologic adaptation of survivors of childhood cancer (mixed‐methods)	Gray et al. (1992) [40]	CA	*N* = 62	F *n* = 22 M *n* = 40	10.7 (NR/1–18)	NR	Unclear	No

Abbreviations: AU, Australia; CA, Canada; F, Female; GB, Great Britain and Northern Ireland; IQR, Interquartile range; JP, Japan; KR, Korea; M, Male; NL, the Netherlands; NR, Not reported; SE, Sweden; US, United States of America.

### 3.3 Methodological Quality of Included Studies

The included studies were of good quality, ranging from 68% to 95% of assigned quality scores, and were all included in the analyses. The weighted Cohen's kappa was 0.90, indicating almost perfect inter‐rater reliability (Supporting Information S1: Appendix D).

### 3.4 How Are Fertility‐Related Concerns Assessed and Defined?

#### 3.4.1 Assessment of Fertility‐Related Concerns

Most studies used qualitative methods to assess fertility‐related concerns, which included interviews (*n* = 11) [11, 13, 22, 23, 25, 27, 28, 31, 33, 34, 38], focus groups (*n* = 3) [25, 29, 30], the analysis of posts in an online discussion forum [19], open‐ended questions as part of a survey [37], and follow‐up essays written by survivors on primary study results [39].

Of the studies that (also) analyzed quantitative questionnaire data (*n* = 10), some used generic late effect/quality of life questionnaires [18, 20, 24, 32, 35, 36, 37, 39, 40], while only one study used a questionnaire specifically designed to assess fertility‐related concerns (Reproductive Concerns After Cancer—Male Scale) [21]. The quantitative instruments and corresponding scales/items are displayed in Table 3.

**TABLE 3:** Quantitative scales and items used to assess fertility‐related concerns.

Instrument [References]	Scales	Items
Impact‐of Cancer‐Childhood Survivor (IOC‐CS) [18, 20, 32]	Relationship concerns Separate items	Worry about telling potential partner about fertility Worry about fertility Worry about my children getting cancer Worry about my children's health
Reproductive Concerns after Cancer ‐Male scale (RCAC‐M) [21]	Fertility potential Achieving pregnancy Acceptance Personal Health Child's Health Partner Disclosure Reproductive Concerns	*N* = 3 items for each subscale
National survey in Japan [24]	Reproductive function and fertility	*N* = 17 items in total
Worry questionnaire by Chesler et al. [35]	Cancer‐specific concerns Present and future concerns	My children getting cancer Whether I can have children
Evaluation sheets completed by psychological team [26]	Psychological health	Fertility concerns
Questionnaire designed by Langeveld et al. [36]	Living situation, marital status, and offspring	Worries about infertility Worries about health of their children
Questionnaire designed by Forsbach & Thompson (mixed‐methods study) [37]	Perceptions of factors that affect adult relationships	Loss of fertility
Questionnaire developed by Weigers et al. (mixed‐methods study) [39]	Experiences, attitudes and needs of survivors (worries)	Able to have children Cancer risk for children
Screening questionnaire from Follow‐Up Clinic (mixed‐methods study) [40]	Psychologic symptoms	Concerns about procreation

Please note that three of these studies used a mixed‐methods approach [37, 39, 40], and one study a combination of qualitative methods [25]. Consequently, these studies included more than one of the aforementioned methods.

#### 3.4.2 Terms and Definitions Used to Describe Fertility‐Related Concerns

**FIGURE 1:**
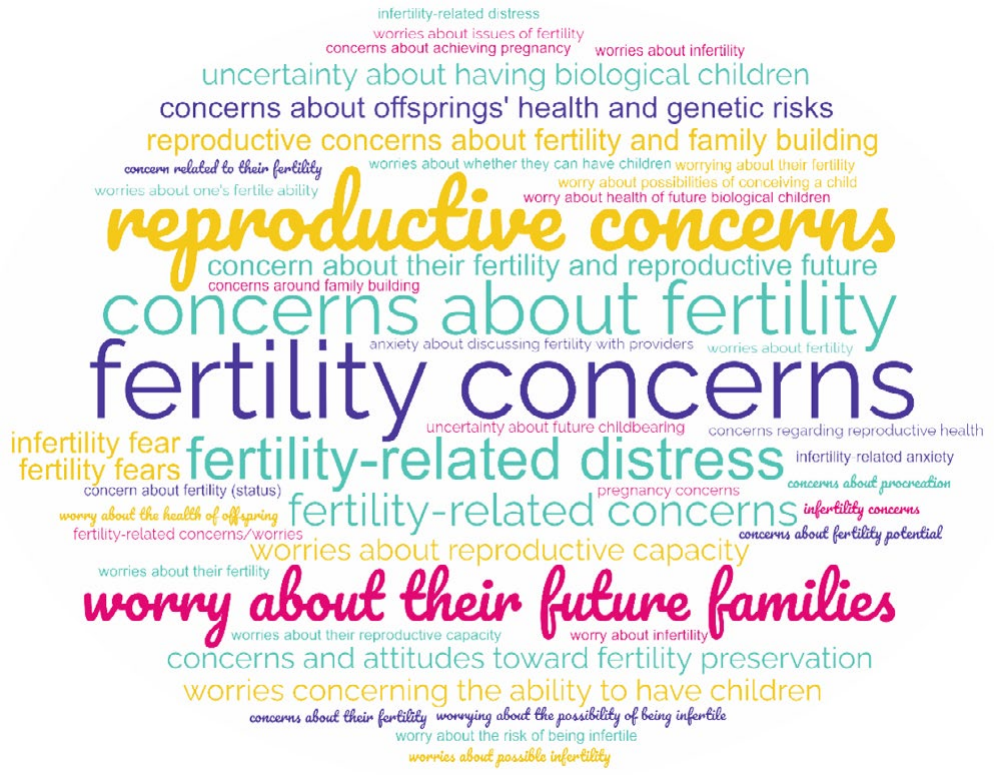
Terms used to describe fertility‐related concerns.

Across publications, we found *N* = 42 different terms to describe fertility‐related concerns (Figure 1). In most publications, different terms were used. The term “fertility concerns” was used in *n* = 8 publications followed by “concerns about fertility” (*n* = 5), “fertility‐related distress” (*n* = 3), and “reproductive concerns” (*n* = 3). The word count of all terms can be found in Supporting Information S1: Appendix E. For the remainder of this paper the term “fertility‐related concerns” will be used to encompass all these terms.

Few studies (*n* = 3) have formally defined fertility‐related concerns. One definition of *reproductive concerns* included “concerns about fertility potential, emotional and practical barriers to achieving pregnancy, worries about one's own physical health affecting capacity to parent, concerns about a possible negative impact of cancer on the health of one's offspring, and worries about disclosing possible infertility to a partner”, in line with the subscales of the Reproductive Concerns After Cancer Scale (RCAC‐M) [21]. Another publication mentioned in the introduction that “unsuccessful attempts to conceive/sterility can cause distress and strain romantic relationships due to frustration, conflicts, and/or break‐ups” [23]. In one qualitative study, the authors coded *fertility or child‐related issues* whenever survivors talked about “the ability to have children, current children, thoughts, concerns, attitudes, or feelings about children in general or about their ability to have children, sexual practices (including ability to have sex, feelings about sex, birth control practices) and their physical condition related to reproductive organs, including uncertainty about their reproductive health” [34].

### 3.5 What Kinds of Fertility‐Related Concerns Do Survivors Describe?

#### 3.5.1 Concerns Related to Personal and Reproductive Health

Survivors described concerns regarding their fertility or potential/possible infertility [19, 21, 35]. Even if they believed that conceiving was possible, female survivors were afraid of having miscarriages or pregnancy complications [11, 19], or that their bodies could not handle the stress and strain of pregnancy [19, 31]. Others were also concerned about being too tired and/or physically unable to take care of a child [19, 32]. In general, survivors were afraid of their overall health, the possibility of a relapse, and an early death (and thus leaving (potential) children behind) [19, 21, 25, 34].

#### 3.5.2 Concerns Related to (Future) Children's Health

When talking about their (future) children, survivors reported fears that their children would be born with a physical, mental, and/or learning disability [31], have health problems [11, 18, 19, 21, 36] or also develop cancer [18, 28]. These concerns were related to their previous treatments or the fear that their cancer might be hereditary.

#### 3.5.3 Concerns Related to Dating and Romantic Relationships

Some survivors described that their unknown fertility status made them shy away from romantic relationships [11]. Others described that their main feelings when meeting and dating potential partners were guilt, shame, and the fear of being a burden, sometimes leading to lowered expectations for marriage partners [28]. Survivors were worried that (potential) infertility would scare off potential partners [22, 38], and they were afraid of disclosing (possible) infertility [18, 21, 32]. Similarly, survivors in a relationship described concerns about discussing fertility issues with their partners [11, 13, 23, 25, 28, 31, 34], sometimes because it had caused problems in past relationships [22, 34]. They were afraid of disappointing their partner [11, 27] or felt bad for them [28].

#### 3.5.4 What Is the Prevalence of Fertility‐Related Concerns Among Childhood Cancer Survivors?

The prevalence of fertility‐related concerns was reported in a total of *n* = 8 qualitative and quantitative studies (Table 4). The prevalence was particularly high in qualitative studies, where concerns were reported by “more than half” [27] and “all but one participant” [31]. In contrast, quantitative studies reported a prevalence ranging from 9.5% to 65%. One study analyzing genders separately indicates that female survivors tend to report more fertility‐related concerns than males [36]. However, another study that collected data from male survivors only had a prevalence rate as high as the one reported by females in the other study [21].

**TABLE 4:** Prevalence of fertility‐related concerns reported in the studies.

Instrument	References	Prevalence
Impact‐of cancer‐childhood survivor (IOC‐CS)	Maas et al. (2023) [18]	> 15%
Impact‐of cancer‐childhood survivor (IOC‐CS)	Van Erp et al. (2021) [20]	48.3%
Reproductive concerns after cancer – male scale (RCAC‐M)	Drizin et al. (2020) [21]	24% high concerns about fertility potential 9.5% high concerns about achieving pregnancy 16.5% low acceptance of infertility 16% high concerns about personal health 44% high concerns about (potential) child's health 27% high concerns about partner disclosure of infertility 61% high concerns on at least one dimension 37% had high concerns on at least two dimensions 21% had high concerns on at least 3 dimensions.
National survey in Japan	Furui et al. (2018) [24]	35.5%
Evaluation sheets completed by psychological team	Gunn et al. (2016) [26]	“Two‐thirds”
Semi‐structured interviews	Frederick et al. (2016) [27]	“More than half”
Semi‐structured telephone interviews	Thompson et al. (2013) [31]	“All but one participant”
Questionnaire designed by Langeveld et al.	Langeveld et al. (2003) [36]	Worries about whether they can have children: Men (53%) Women (65%) Worries about children getting cancer: Men (40%) Women (51%)

### 3.6 What Are the Clinical Implications for Follow‐Up Care?

#### 3.6.1 Addressing Fertility‐Related Concerns in Follow‐Up Care

Survivors expressed an urgent need for information and education regarding their fertility status and any related concerns [11, 24, 28, 33, 38]. Besides such information provision/education, practical support might be especially important [13]. In addition to fertility, providers should address topics related to identity, love/romance, relationship building, partner communication, sexuality, and body image [13, 22, 26, 27, 31] as these have been identified as important key factors related to fertility‐related concerns.

While most recommendations emphasized individual discussions with survivors, others also emphasized the potential benefit of peer support in the form of group sessions or discussion forums, as survivors could appreciate the guidance and support of other survivors [13, 19]. This could provide an opportunity for communication, a sense of belonging, and a way to normalize talking about potentially sensitive topics.

#### 3.6.2 Assessing Individual Perspectives and Attitudes

When addressing fertility‐related topics in survivorship care, there are several factors to consider that may influence survivors' attitudes toward the topic. Factors that were mentioned across studies to be considered were age and developmental stage [13], sexual orientation/identity [11, 22], sexual (in‐) experience [22], reproductive goals and the option of not wanting to have children [23, 34], cultural background, and stigma related to cancer [28], or religious beliefs against fertility preservation [33]. It is important that providers explore perceptions and needs by *asking* survivors (and parents) individually instead of making assumptions [29, 33].

Survivors expressed wanting a choice regarding whether or not parents should be involved in fertility‐related discussions [33]. If fertility‐related discussions only involved parents at the time of diagnosis, this could lead to later regret in survivors and the feeling that their needs were being marginalized by providers [11, 33]. Furthermore, parents may want to protect their children and therefore withhold information [34], which can lead to later uncertainty and concerns. However, parents, and sometimes older siblings and grandmothers, were also identified as sources of emotional and practical support [33].

Several studies suggested different types of providers that should be involved in fertility‐related discussions/support, which included nurses [11, 13], counselors [11], psychologists with fertility‐related expertise [11], pediatric oncologists [27], fertility specialists [29], or multidisciplinary healthcare teams in general [13, 29]. One study found that involving only one or two professionals and a small number of family supporters in these discussions worked well [33].

#### 3.6.3 Onset and Regularity of Fertility‐Related Discussions

Many authors recommend addressing fertility‐related topics around diagnosis as early as possible [13, 26, 27, 28, 29, 33]. Although this recommendation is likely based on offering patients the option of fertility preservation prior to treatment, survivors in one study explicitly stated that fertility should be addressed at the time of diagnosis, even if preservation is not available or appropriate [33]. Other authors state that access to support is especially important early in the survivorship journey [11]. In another study, survivors said that providers should consider their age and developmental stage when initiating discussions [27].

Most authors recommend that fertility issues should be addressed systematically and regularly throughout survivorship [11, 13, 21, 22, 23, 25, 26, 30]., as reproductive goals may change over time [23].

## 4 Discussion

This systematic review demonstrated considerable variation in the assessment, terminology, and definition of fertility‐related concerns in childhood cancer survivors. Based on this evidence, we propose to define fertility‐related concerns as a wide range of psychosocial concerns, including but not limited to concerns about fertility potential, generic and reproductive health, the health of possible (future) children, and dating and romantic relationships. The prevalence of concerns varied with up to 65% of survivors reporting fertility‐related concerns (in quantitative studies). Clinical implications of the included studies suggest that different healthcare providers could address fertility‐related concerns repeatedly into long‐term survivorship. They should focus on patients/survivors rather than parents and assess individual preferences and attitudes without making assumptions about fertility‐related goals and attitudes.

Our results show that there is currently no consistent understanding of fertility‐related concerns in the context of childhood cancer survivorship. A total of *N* = 42 terms were found to describe the concept, including concern, worry, fear, anxiety, and distress, which were used interchangeably.

To properly measure and ultimately address fertility‐related concerns in research and practice, we believe that it is essential to develop a concise and corresponding definition, as is the case with other psychological late effects. *Fear of cancer recurrence*, for example, is a fixed term in the literature and usually defined as “the fear that cancer could return or progress in the same place or another part of the body” [41]. Similarly, *cancer‐related fatigue* is constantly used and defined as “a distressing, persistent, subjective sense of tiredness or exhaustion related to cancer or cancer treatment that is not proportional to recent activity and interferes with usual functioning.” [42] A comparable definition and fixed term would be desirable for fertility‐related concerns. It would create a common language for researchers, practitioners, and survivors affected by fertility‐related problems and concerns. The standardization of terms could reduce misunderstandings and facilitate effective communication. It would also allow for comparisons to be made between studies and populations.

Considering survivors' descriptions of fertility‐related concerns, the main areas of concern were their personal and reproductive health, the health of their (future) children, and concerns about dating and relationships. However, the concerns expressed likely depended on how they were assessed. The complexity of survivors' concerns suggests that assessment tools must be quite broad to adequately cover the different domains. Although interviews are a well‐established means of obtaining a comprehensive picture of a topic for research purposes, they are difficult to use in everyday clinical practice because they are very time‐consuming. The Reproductive Concerns After Cancer Scale (RCACS) is a multidimensional scale that uses 18 items to measure concerns about fertility potential, partner disclosure, child's health, personal health, acceptance, and pregnancy, with versions for female [43] and male cancer survivors [44]. The RCACS is a reliable and valid measure with promising potential for use in research *and* clinical practice [43, 44]. While the studies included in this review provide important information to better understand the various fertility‐related concerns of childhood cancer survivors, it would be desirable to explore comprehensive and standardized measurement tools as a future step, as most research to date has been done on adolescent and young adult cancer patients and survivors. This would facilitate the comparison of prevalence in different samples, which is currently difficult, as can be seen from our results, where prevalence ranged from 9.5% to 65%.

### 4.1 Clinical Implications

Although the prevalence of fertility‐related concerns has not been consistently reported, our results suggest that they are common in childhood cancer survivor populations. Providers should assess these concerns systematically and repeatedly to address them appropriately. Importantly, assessment of fertility‐related concerns should occur regardless of presumed fertility status and be repeated due to young survivors' development over time. Healthcare providers may broach the topic of fertility by openly asking about reproductive goals and further assessing survivors' fertility‐related knowledge to further tailor fertility‐related discussions with individual patients. Such tailored discussion may further be guided by inquiring about common fertility‐related concerns (e.g., Do you sometimes worry about …) and include psychoeducation, offering fertility assessments (if indicated). Comprehensive tools such as the RCACS(−M) [43, 44] may be appropriate to discuss fertility‐related concerns in depth.

By addressing these complex concerns, healthcare providers could empower survivors in their family‐building options, enhancing their well‐being and improving their quality of life after treatment. Beyond educating survivors about their fertility, emotional and practical support is essential, addressing issues such as identity, love, relationship dynamics, partner communication, sexuality, and body image. It is important that providers address fertility‐related concerns without making assumptions about survivors' attitudes. Engaging a broad spectrum of stakeholders, including survivors and their families, in conversations about fertility is crucial for creating care models that are inclusive and tailored to address the distinct needs of all survivors. The influence of the presence of parents in discussing fertility‐related concerns is an important aspect, particularly during the time of treatment in childhood and adolescence. Even throughout survivorship and when survivors become legal adults, they may still be accompanied by parents to follow‐up visits, and conversations may become more challenging when discussing sensitive topics like fertility, relationships, and family‐building, which may feel uncomfortable. Therefore, providers are advised to have individual discussions with survivors of legal age about sensitive and intimate topics. In addition to individual counseling, survivors might benefit from group peer support sessions or discussion forums.

When addressing survivors' concerns, medical aspects of their fertility should also be highlighted. Survivors' fertility‐related concerns may or may not be rooted in actual knowledge about their fertility status. Unfortunately, childhood cancer survivors are often unaware of their health risks, including fertility issues, or receive information too late [45, 46, 47]. Studies have shown that many adult survivors express a desire to have children later in life but are often uninformed about their fertility status [12]. This lack of knowledge can lead to delays in seeking appropriate medical advice or interventions, which may affect their ability to conceive or have children at all. Addressing this issue requires proactive efforts by healthcare providers to educate survivors about their potential infertility risks and fertility preservation options, empowering them to make informed decisions about their reproductive health.

Importantly, in many countries, national or local guidelines recommend including fertility and sexual health concerns in long‐term follow‐up care. Local guidelines can serve as valuable tools for providers seeking structured approaches to sexual health in survivorship care. Several previous publications have also summarized actionable recommendations that clinicians can use to better support survivors in this area [48, 49, 50].

### 4.2 Study Limitations

Despite the careful development of the search strategy, we found more than half of the included studies through reference list screening. The lack of a clear definition of fertility‐related concerns added to the challenge of designing our search, but also presented one goal of this research: to gain a better understanding of fertility‐related concerns. Moreover, included studies were conducted in Western countries, often in high‐resource settings, with almost half of them being conducted in the United States. Therefore, the results cannot be generalized to all geographic contexts, as cultural aspects and healthcare systems differ.

Future research on fertility‐related concerns in childhood cancer survivors should focus on several key areas: First, there is a need for a standardized definition and consistent terminology to enhance measurement and facilitate comparisons across studies. Second, developing and validating comprehensive assessment tools, like the RCACS, for diverse survivor populations and assessments sensitive to the development of young survivors over time is essential. Additionally, research should examine the role of sociodemographic and cultural factors in shaping survivors' concerns, with an emphasis on including more diverse, international samples. Our review revealed substantial variability in the reported prevalence of fertility‐related concerns, but the underlying reasons for this variability remain unclear. Factors such as differences in assessment tools, gender, and cultural background may play a role, underscoring the need for in‐depth investigations of reasons for variability. Longitudinal and intervention studies are also needed to evaluate how fertility concerns evolve and to test the effectiveness of various support strategies. Integrating fertility‐related discussions into routine clinical practice and understanding how healthcare providers can address these concerns proactively would improve survivorship care and fertility‐related decision‐making. Finally, a particularly important area for future research is the impact of fertility preservation techniques on survivors' fertility‐related concerns. As these techniques become more common, especially in the past two decades, it would be valuable to explore whether their availability has shifted the nature or prevalence of these concerns over time.

The strengths of this review lie in the inclusion of studies conducted over the past 35 years, using a variety of methodologies to address the question at hand. We sought to minimize the risk of missing important publications on the topic through reference screening. Our analysis provides a comprehensive overview of the assessment and definition of fertility‐related concerns, encompassing the multiple concerns expressed by survivors and the clinical implications proposed by the authors. In addition, the significance of our findings is underscored by the substantial participation of over 4200 survivors across all included studies.

## 5 Conclusions

Fertility‐related concerns in survivors of childhood cancer include a wide variety of psychosocial concerns about fertility potential, general and reproductive health, the health of possible (future) children, and dating and romantic relationships. These concerns are common among childhood cancer survivors and deserve to be addressed by providers and researchers in the field. To do so effectively, we need more consistency in how these concerns are measured, labeled, and defined.

## Author Contributions

P.H., G.M., V.L., and F.S.M.S. contributed to the review design and secured funding for the study. P.H. searched the literature, P.H. and M.O. double‐screened all references, P.H. extracted the data, M.O. double‐checked the data extraction, and P.H. and M.O. assessed the quality of the included studies. P.H. wrote the initial draft of the manuscript, and all authors provided feedback on it. All authors read and approved the final manuscript.

## Conflicts of Interest

The authors declare no conflicts of interest.

## Supporting information


Data S1.

## Data Availability

The data that support the findings of this study are available from the corresponding author upon reasonable request.
